# Some Dangers of Spoon-Feeding

**Published:** 1913-03-01

**Authors:** 


### SOME DANGERS OF SPOON-FEEDING.

Indirectly the question raised by Mr. Geoffrey Drage, and supported by the *Spectator* a month ago, relates to the dangers of spoon-feeding a nation. Mr. Drage properly insists that the Government should order a return to be prepared of the total expenditure out of national and out of local funds upon what the French call "public assistance," but what in effect in this country is mainly State charity. During the last twenty-five years the growth of this State charity has so increased in Great Britain that it has come to supersede in importance the Poor-Law organisation, which, as well as the very able reports of the late Commission, has been entirely ignored by the present Government, which has belittled the President of the Local Government Board and his Department, despite the increase in the salary of Mr. John Burns, and increased enormously the burden of State charity in these islands. We are quite aware that Mr. John Burns has quietly and courageously borne the slights which have been cast upon him and his Department, and that he has steadily endeavoured to show, with excellent results, how much good improved administration can effect in various directions. But the fact remains, so little interest do the people seem to take in the reform of the Poor Law, so utterly have these reforms found themselves at a discount so far as the present Government is concerned, that nothing has come out of the Poor-Law Commission's Report, and the old evils are allowed to continue and increase.

If Mr. John Burns would bring in a Bill like that we have suggested elsewhere to deal with Weary Willies and loafers at the present time, he would do much to awaken and fix public interest upon a problem which must be faced and solved. Then, providing in addition some active members of Parliament would make it their business to obtain from the Government the return suggested by Mr. Drage, public attention would be aroused and held, because the people would thus be made cognisant of the aggregate cost of our State charities each year. The return, to be effective, must be annual, and it must show "the number of persons in receipt of public assistance in all forms on a given date," together with "the total expenditure for the whole year on service of the class in question, distinguishing salaries and administrative expenses, and also setting forth the amounts raised by the rates or contributed by the taxpayer."

State charity at present includes a very large expenditure under the plea of finding employment for the unemployed, though the evils attaching to this plan were exposed eighty years ago in the famous Report of the Poor-Law Commissioners in 1834. So that in this return must be included all schemes for providing employment through the municipal authorities, the cost of Old Age Pensions, the cost of free education, of national insurance so far as it is subsidised out of the pocket of the taxpayer, the cost of maintenance in isolation hospitals, and the upkeep and cost of pauper lunatics over and above the contribution of their friends. It must further embrace the amount spent on free meals for school children and the total cost of the subsidy granted out of the rates in the form of workmen's tickets for tramways. Further, this return, to be effective, must show clearly the cost under each special head, which should be distinctly set out.

At present State charity expenditure is so muddled up in the returns made by a number of different departments, national and local, as to prevent anyone from obtaining accurate knowledge of the whole outlay. The truth is England is becoming more and more every day a spoon-fed nation. To spoon-feed a people is to demoralise and degrade them. We should be glad to open our columns to a correspondence dealing with these questions from every point of view. The sooner and fuller they are ventilated and brought home to the minds and consciences of all thoughtful people the better will it be for each individual and for the whole of the people resident within the British Isles.

